# Structured Versus Non‐Structured Reporting of Inflammatory Bowel Disease Imaging: A Systematic Review

**DOI:** 10.1002/jgh3.70288

**Published:** 2025-09-28

**Authors:** Richard Lo, Ziang Ma, Lynna Chen, Abhinav Vasudevan, Ashish Srinivasan

**Affiliations:** ^1^ The University of Melbourne Department of Medicine, Austin Health Melbourne Australia; ^2^ Eastern Health Department of Gastroenterology Melbourne Australia; ^3^ Monash University, Eastern Health Clinical School Melbourne Australia; ^4^ Austin Health Department of Gastroenterology Melbourne Australia

**Keywords:** computed tomography, inflammatory bowel disease, magnetic resonance, radiology, structured reporting

## Abstract

**Background:**

Effective communication between radiologists and clinicians is essential for optimal inflammatory bowel disease (IBD) management. Structured reporting (SR) of imaging reports may enhance interdisciplinary communication and clinical decision‐making; however, its utility compared to non‐structured reporting (NSR) in IBD remains unclear. This systematic review evaluated IBD clinician perceptions of SR versus NSR in IBD‐related imaging.

**Methods:**

Embase, MEDLINE, and CENTRAL were searched to January 2025 for studies comparing SR and NSR in abdominal and pelvic imaging for IBD, including magnetic resonance imaging (MRI), computed tomography (CT), and intestinal ultrasound (IUS). The primary outcome was perceived clarity and clinical utility by the referring clinician, with report completeness evaluated as a secondary outcome.

**Results:**

Six studies met inclusion criteria, comprising 199 IBD patients and 224 scans (105 MRI, 119 CT), with a total of 550 SR/NSR report pairs evaluated by 19 clinicians. No eligible studies assessed pelvic MRI or IUS. In four of five studies, clinicians perceived SR as clearer than NSR. Similarly, SR were viewed as having greater clinical utility for assessing disease activity, identifying disease phenotype, and influencing management decisions in four studies. SR were also associated with more complete reporting based on predefined radiological criteria in three studies.

**Conclusion:**

Clinicians generally perceived SR to provide greater clarity and clinical utility than NSR in IBD‐related imaging, potentially enhancing interdisciplinary communication and clinical decision‐making. Further research is needed to validate these findings and evaluate their impact on patient outcomes in routine IBD practice.

## 1 Introduction

Magnetic resonance imaging (MRI), computed tomography (CT), and intestinal ultrasound (IUS) are important cross‐sectional imaging modalities used in the diagnosis and monitoring of inflammatory bowel disease (IBD) [1]. While MRI and CT are typically interpreted by radiologists, IUS may be performed and reported by gastroenterologists or radiologists. IBD specialists rely on imaging reports to inform therapeutic decision‐making, particularly in defining disease extent and severity, identifying disease‐related complications, and undertaking assessments of treatment response. This reflects the significance of radiology report structure and content in facilitating effective interdisciplinary communication between radiologists, bowel sonographers, and the broader IBD care team [2, 3].

Despite their central role in clinical care, radiology reports vary considerably in structure and content across institutions and between practitioners [4, 5]. The traditional non‐structured report (NSR), a free‐text format without standardized content, remains the most common radiological reporting method in IBD. While NSR offers flexibility, it is susceptible to inconsistencies, omissions, and ambiguous language, which may contribute to miscommunication, misinterpretation, and suboptimal clinical decision‐making [6]. In the context of multidisciplinary IBD care, such variation may contribute to inconsistent care delivery, a recognized barrier to optimal IBD outcomes [7].

Structured reporting (SR) has emerged as an alternative format of radiology reporting that uses reproducible, template‐based frameworks with predefined required and optional elements. SR promotes consistency, improves report completeness, and facilitates clearer communication between radiologists and referring clinicians [6, 8, 9]. Consequently, SR has been widely adopted in oncologic radiological reporting, where standardized reporting remains essential for accurate staging and treatment planning [10, 11]. However, the application of SR in non‐malignant conditions, including IBD, has been comparatively limited. This may be attributed to concerns about reduced flexibility when reporting complex diseases, potential workflow inefficiencies, and inconsistent evidence regarding the benefits of universal adoption of SR formats [12, 13, 14, 15].

Given the chronic, relapsing nature of IBD and the need for serial imaging, a more structured approach to radiology reporting may improve interdisciplinary communication, reduce diagnostic ambiguity, and facilitate optimal clinical decision‐making [16, 17, 18, 19]. Recognizing this, expert bodies such as the European Crohn's and Colitis Organization–European Society of Gastrointestinal and Abdominal Radiology (ECCO–ESGAR) and the Society of Abdominal Radiology Crohn's Disease Focused Panel, Society of Pediatric Radiology, and American Gastroenterological Association (SAR CDFP‐SPR‐AGA) have developed consensus recommendations for radiology reporting in IBD (Table 1) [16, 17, 18, 19]. Despite these efforts, SR templates associated with MRI, CT, and IUS have not been widely adopted in IBD imaging practice. This systematic review aims to evaluate the perceptions of referring clinicians with respect to SR versus NSR of IBD‐related imaging.

**TABLE 1:** Key radiological features to consider when reporting inflammatory bowel disease activity and complications, based on current guidelines.

	SAR CDFP‐SPR‐AGA (2018) [19]	ECCO‐ESGAR (2022) [16]
Indication	Crohn's disease	IBD
Imaging modality	MRI, CT	MRI, CT, IUS
Mural		
Thickening	✓	✓
Oedema	✓	✓
Ulceration	✓	✓
Enhancement pattern	✓	
Extramural		
Mesenteric oedema	✓	✓
Comb sign	✓	✓
Fibrofatty proliferation	✓	✓
Mesenteric lymphadenopathy	✓	✓
Mesenteric vein thrombosis	✓	✓
Sacculation	✓	
Intestinal hyperaemia		✓
Extra‐intestinal		
Perianal disease	✓	✓
Sacroiliitis	✓	
Avascular necrosis	✓	
Primary sclerosing cholangitis	✓	
Cholelithiasis	✓	
Nephrolithiasis	✓	
Complications		
Sinus tract	✓	✓
Fistula	✓	✓
Abscess	✓	✓
Inflammatory mass	✓	✓
Perforation	✓	✓
Stricture	✓	✓
Stricture upstream dilatation	✓	✓
Neoplasm		✓
Technical aspect		
Diffusion restriction	✓	✓
Reduced motility	✓	✓
Other		
Disease location	✓	✓
Number of disease segments	✓	✓
Disease length	✓	✓
Therapy response	✓	✓
Post‐operative disease activity		✓
Radiological disease activity score		✓

Abbreviations: AGA, American Gastrointestinal Association; CT, computed tomography; ECCO, European Crohn's and Colitis Organization; ESGAR, European Society of Gastroenterology and Abdominal Radiology; IUS, intestinal ultrasound; MRI, magnetic resonance imaging; SAR CDFP, Society of Abdominal Radiology Crohn's Disease‐Focused Panel; SPR, Society of Pediatric Radiology.

## 2 Methods

This systematic review was conducted in accordance with the Preferred Reporting Items for Systematic Reviews and Meta‐Analyses (PRISMA) recommendations for the reporting of systematic reviews and was prospectively registered on Prospero (CRD420251038937) [20].

### 2.1 Data Sources and Search Strategy

A comprehensive search of EMBASE, MEDLINE, and Cochrane Central Register of Controlled Trials (CENTRAL) databases, from inception to January 2025, was performed to identify eligible studies. The full search strategy, including keywords and operators used, is shown in Table S1.

### 2.2 Inclusion and Exclusion Criteria

Studies eligible for inclusion in this systematic review included clinical trials (non‐randomized or randomized) and observational studies (prospective or retrospective) that included (1) patients with IBD who required (2) cross‐sectional abdominal or pelvic imaging using MRI, CT, or IUS where (3) SR was compared with NSR, and (4) the primary outcome of perceived clarity or clinical utility from referring clinicians was reported. Studies that included mixed cohorts, that is, cohorts that included patients with and without IBD, were only eligible for inclusion when outcomes specific to patients with IBD could be clearly delineated. Other exclusion criteria were unpublished studies and studies not published in English.

### 2.3 Outcomes

The primary outcome was to assess the perceived clarity and clinical utility of SR compared to NSR in cross‐sectional imaging for IBD evaluation. The secondary outcome was the completeness of radiology reports.

### 2.4 Definitions

SR was defined as any radiology reporting method that utilized a predefined template, specified the organization of the radiology report, outlined required radiological content, or included a checklist of key radiological items to be reported. NSR was defined as any reporting structure that did not meet the criteria for SR and was generally defined as those that did not make any specification regarding report content or structure. The referring clinician was defined as the medical practitioner who requested cross‐sectional imaging and included medical and surgical clinicians. Clarity was defined as the degree to which key radiological features relevant to IBD were clearly conveyed and easily extractable from the radiology report. The clinical utility of the reporting structure was defined as the perceived benefit in identifying disease activity, characterizing disease phenotype, or influencing clinical decision‐making. Report completeness was assessed through objective evaluation of the presence of radiology content against predefined radiological parameters.

### 2.5 Study Selection and Data Extraction

Two authors (RWL, ZM) independently screened eligible study titles and abstracts identified by the search and excluded those that were unrelated based on the pre‐specified inclusion and exclusion criteria. This process was undertaken with the assistance of the Covidence software program [21]. The full text of selected articles was appraised to determine suitability for inclusion, with conflicts in study selection resolved by consensus in consultation with a senior investigator (ARS). The electronic search was supplemented by a manual review of conference abstracts and the citation list of included studies. The following data were extracted from eligible studies: first author name, year of publication, country of publication, study design, number of patients with IBD, patient characteristics, IBD subtype, imaging modality, manner of radiological reporting (NSR or SR), and primary and secondary outcomes of interest. A meta‐analysis was not conducted due to expected methodological heterogeneity and an emphasis on descriptive, rather than quantitative, assessment of mixed methods outcome reporting.

### 2.6 Risk of Bias Assessment

The methodological quality of the included studies was assessed by two authors (RWL, ZM). The Joanna Briggs Institute (JBI) critical appraisal tool for quasi‐experimental study was used to evaluate the risk of bias of the six included studies [22]. Studies were graded as high, moderate, or low quality based on an overall score of > 75%, ≤ 75% and ≥ 50%, or < 50%, respectively. Conflicts were resolved by consensus. Only studies that scored high or moderate quality were included.

## 3 Results

### 3.1 Search Results

**FIGURE 1:**
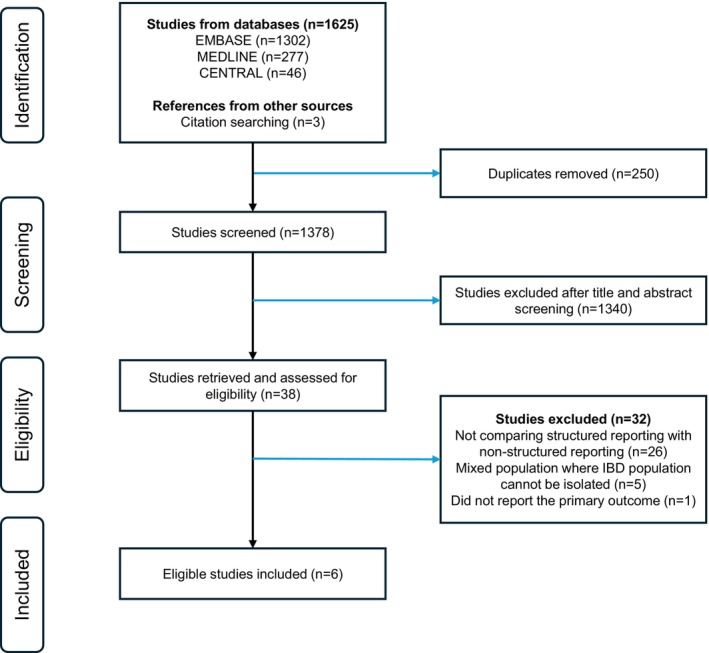
Preferred reporting items for systematic reviews and meta‐analyses (PRISMA) flow diagram.

The search strategy identified 1625 studies, with three additional studies identified through citation tracking. After removing 250 duplicates, 1378 unique records were screened by title and abstract. Thirty‐eight articles were identified for full‐text review, with six studies identified to meet all eligibility criteria (Figure 1).

### 3.2 Risk of Bias Assessment

Study quality was evaluated using the JBI critical appraisal tool. All six included studies were nonrandomized observational studies, comprising four full‐text publications and two conference abstracts. Based on the overall appraisal scores, two studies were rated as high quality, while the remaining four were assessed as moderate quality (Table S2).

Both studies conducted by Wildman et al. were designated to be of high quality, each receiving an overall JBI score of 87.5% (7/8). Apart from the concern for study reliability from the lack of referring clinician and radiologist blinding, the methodologies of both studies fulfilled all other JBI criteria [23, 24]. The studies by Chiplunker et al. and Gomez et al. were both appraised to be of moderate quality, each receiving overall scores of 62.5% (5/8). The studies by Chiplunker et al. and Gomez et al. were both appraised to be of moderate quality with overall scores of 62.5% (5/8). Importantly, neither study specified details of the number and experience of radiologists involved in generating the SR and NSR. Similarly, the lack of referring clinician and radiologist blinding reduced the reliability of both studies [25, 26]. The study by Bailey et al. was appraised to be of moderate quality with an overall JBI score of 50% (4/8). Although the radiologists interpreting the SR were appropriately blinded, the radiologists involved in the interpreting the NSR were not. Further, the referring clinicians were not blinded and the statistical analyses employed in this study were not explicit [27]. The study conducted by Zhu et al. was appraised to be of moderate quality with an overall score of 55.5% (5/9). It was important to note that the NSR and SR were interpreted by radiologists of varying levels of experience, without evidence of calibration, which may have contributed to heterogeneity in reporting practices. The lack of referring clinician and radiologist blinding also affected study reliability. In addition, missing details, including the number of participants lost to follow‐up during the study, further limit the overall quality of this longitudinal study.

### 3.3 Study Characteristics

Five of the six included studies were retrospective in design, with the final study incorporating retrospective and prospective elements (Table 2). Collectively, these studies included 199 patients with IBD, including 114 with Crohn's disease, three with ulcerative colitis, and two with IBD‐unclassified (IBD‐U). The IBD subtype was not specified in the remaining 80 patients. A total of 224 cross‐sectional imaging examinations were performed across the six included studies, comprising MR enterography (MRE, *n* = 105) and CT enterography (CTE, *n* = 119), with 550 NSR and 550 SR compared. None of the included studies appraised SR and NSR related to IUS or pelvic MRI.

**TABLE 2:** Studies comparing structured versus non‐structured imaging reports in inflammatory bowel disease.

Authors (Year) Study design	IBD subtype (*n*)	Imaging modality (*n*)	SR/NSR	Referring physicians/surgeons/radiologists (*n*)	Referring clinicians' perception (SR vs. NSR)	Guideline‐based template	Completeness of report (SR vs. NSR)
Wildman‐Tobriner et al. [24] (2017) Retrospective Prospective	Crohn's disease (25)	MRE (25)	75/75	2/1/3	Information extraction (1.7 vs. 1.2, *p* < 0.05) Anatomical clarity (1.9 vs. 1.4, *p* < 0.05)^a^ Ability to identify disease phenotype (1.7 vs. 1.1, *p* < 0.05)^a^	Yes SAR CDFP (2015) [18]	SR > NR: (14.0 vs. 7.7, *p* < 0.05)
Wildman‐Tobriner et al. [23] (2017) Retrospective	Crohn's disease (25) Ulcerative colitis (3) IBD‐unclassified (2)	CTE (30)	270/270	2/1/9	Information extraction (1.7 vs. 1.2, *p* < 0.05)^a^ Anatomical clarity (1.8 vs. 1.7, NS)^a^ Ability to identify disease phenotype (0.98 vs. 0.87, *p* = 0.05)^a^	Yes SAR CDFP (2015) [18]	SR > NR (14.6 vs. 8.2, *p* < 0.05)
Chiplunker et al. [25] (2018) Retrospective	Crohn's disease (25)	MRE (50)	50/50	2/0/—	Identifying active disease (100 vs. 63%, *p* < 0.05) Identifying stricturing disease (97 vs. 47%, *p* < 0.05) Change management (77 vs. 48%, *p* < 0.05)	Yes SAR CDFP‐SPR‐AGA (2018) [19]	—
Gomez et al. [26] (2018) Retrospective	Crohn's disease (9)	CTE (9)	45/45	2/3/—	Clarity (8.5–9.25 vs. 7.75–8.5, *p* < 0.05) Defining IBD extent (8.5–9.25 vs. 7.75–8.5, *p* < 0.05) identifying IBD complications (9.25–10 vs. 7.75–8.5, *p* < 0.05) Determine IBD behavior (8.5–9.25 vs. 7.75–8.5, *p* < 0.05) Identifying disease activity: (8.5–9.25 vs. 7.75–8.5, *p* < 0.05)	No	—
Bailey et al. [27] (2021) Retrospective	IBD (80)	MRE (30) CTE (50)	80/80	3/0/2	Clarity (9.9 vs. 7.6, *p* < 0.05) Efficiency of information extraction: (9.9 vs. 7.6, *p* < 0.05) Management change for 20/80 patients (25%)	No	SR > NR (77/80 vs. 40/80 reports, *p* not provided)
Zhu et al. [28] (2024) Retrospective	Crohn's disease (30)	CTE (30)	30/30	2/1/8	Clarity: (4.20 v 3.43, *p* < 0.05)	Yes SAR CDFP‐SPR‐AGA (2018) [19]	—

*Note:* NS: not significant *p* > 0.05**;** —: unknown.

Abbreviations: AGA, American Gastrointestinal Association; CTE, computed tomography enterography; IBD, inflammatory bowel disease; MRE, magnetic resonance enterography; NSR, non‐structured reporting; SAR CDFP, Society of Abdominal Radiology Crohn's Disease‐Focused Panel; SR, structured reporting; SPR, Society of Pediatric Radiology.

^a^ 15 SR versus 15 NSR were evaluated.

### 3.4 Study Outcomes

Referring clinicians' perceptions of SR versus NSR in the context of cross‐sectional imaging for IBD were evaluated across several domains, including clarity (*n* = 5) and clinical utility (*n* = 4) [23, 24, 25, 26, 27, 28]. Report completeness was assessed by three studies [23, 24, 27]. The perceptions of 19 medical (*n* = 13) and surgical (*n* = 6) referring clinicians were evaluated across the included studies, with each study appraising the views of between two and five clinicians [23, 24, 25, 26, 27, 28].

All six studies defined SR as a radiology report generated using a standardized reporting template that required the inclusion of specific content and/or key radiological parameters [23, 24, 25, 26, 27, 28]. Four studies referenced the SAR CDFP consensus guidelines [18, 19, 23, 24, 25, 28]. One study employed the reporting template from a published conference presentation [26, 29]. One study developed the reporting template based on a literature review [27]. In contrast, NSR was defined in only three of six studies [23, 24, 28]. Two studies defined NSR as a free‐text report without specific reporting criteria, while the third described it as a radiology report lacking predefined structure and content guidelines.

### 3.5 Perceptions of the Referring Clinician

#### 3.5.1 Perceived Clarity

The perceived clarity of cross‐sectional imaging reports was evaluated qualitatively in five studies using clinician surveys [23, 24, 26, 27, 28]. Only one study provided a clear definition of “clarity” for respondents. Wildman‐Tobriner et al. compared 15 SR and 15 NSR from MRE scans in patients with IBD. Three clinicians rated these reports, with SR appraised to be significantly clearer in communicating patient anatomy (mean score on a 0–2 scale: 1.9 vs. 1.4, *p* < 0.05) [24]. Similarly, Gomez et al. found higher clarity ratings for SR in a study involving 45 SR and 45 NSR from 9 MRE scans. Two gastroenterologists and three colorectal surgeons evaluated the reports, again showing a significant advantage for SR (mean score on a 0–10 scale: 8.5–9.25 vs. 7.75–8.5, *p* < 0.05) [26]. Bailey et al. was the only study to explicitly define “clarity,” describing it as the degree to which key radiological features were clearly conveyed. In this study, three gastroenterologists rated 80 SR and 80 NSR across 30 MRE and 50 CTE, finding that SRs scored significantly higher in clarity (median score on a 1–10 scale: 9.9 vs. 7.6, *p* < 0.05) [27]. Zhu et al. found consistent results: three clinicians assessed 30 SR and 30 NSR from 30 CTE studies, with SR again scoring higher on perceived clarity (median score on a 1–5 scale: 4.2 vs. 3.43, *p* < 0.05) [28]. In contrast, a subsequent study by Wildman‐Tobriner et al. found no significant difference in clarity between 15 SR and 15 NSR for CTE scans (mean [0–2 scale]: 1.8 vs. 1.7, *p* > 0.05) [23].

Three studies appraised perceived clarity in terms of ease of extracting relevant information from cross‐sectional imaging reports related to MRE and/or CTE [23, 24, 27]. Only Bailey et al. defined this outcome explicitly as the efficiency with which key information could be retrieved from radiology reports [27]. Wildman‐Tobriner et al. found it significantly easier to extract information from SR than NSR across two separate studies: one involving three clinicians evaluating 15 SR and 15 NSR for MRE scans (mean [0–2 scale]: 1.7 vs. 1.2, *p* < 0.05), and another with three clinicians assessing 15 SR and 15 NSR for CTE scans (same scoring, *p* < 0.05) [23, 24]. Bailey et al. reported similar findings among three gastroenterologists assessing 160 reports (80 SR and 80 NSR) for both MRE and CTE, documenting a clinician preference towards SR (median [1–10 scale]: 9.9 vs. 7.6, *p* < 0.05) [27].

#### 3.5.2 Perceived Clinical Utility

The perceived ability to identify disease phenotype from imaging reports was assessed in four of five eligible studies using clinician surveys [23, 24, 25, 26]. Of these, only one provided a definition, describing it as the clinician's confidence in identifying Crohn's disease phenotypes [25]. In two studies by Wildman‐Tobriner et al., three referring clinicians evaluated 15 SR and 15 NSR for MRE and CTE imaging, respectively. SR templates were rated significantly higher for their ability to communicate IBD phenotype (mean [0–1 scale]: 1.0 vs. 0.8 for MRE, *p* < 0.05; 0.98 vs. 0.87 for CTE, *p* = 0.05) [23, 24]. Chiplunker et al. reported that two IBD‐focused gastroenterologists rated SR as providing more confidence than NSR in identifying active Crohn's disease (100% vs. 63%, *p* < 0.05) and stricturing disease (97% vs. 47%, *p* < 0.05) across 50 SR and 50 NSR for 50 MRE scans [25]. Similarly, using the mean score on a 0–10 scale, Gomez et al. found that two gastroenterologists and three colorectal surgeons rated SR as being better than NSR in identifying IBD disease extent (8.5–9.25 vs. 7.75–8.5, *p* < 0.05), disease behavior (8.5–9.25 vs. 7.75–8.5, *p* < 0.05), disease activity (8.5–9.25 vs. 7.75–8.5, *p* < 0.05), and disease complications (9.25–10 vs. 7.75–8.5, *p* < 0.05) across 45 SR and 45 NSR for 9 MRE scans [26].

The influence of SR on clinical decision‐making was evaluated in two of six eligible studies [25, 27]. Chiplunker et al. reported that two IBD gastroenterologists reviewed 50 SR and 50 NSR for 50 MRE, identifying significantly more management changes to pre‐existing treatment plans based on SR compared to NSR (77% vs. 48%, *p* < 0.05) [25]. Similarly, Bailey et al. reported that SR (*n* = 80) for 30 MRE and 50 CTE scans would have led to a change in clinical management in 25% of cases (20 patients) based on a retrospective assessment by three gastroenterologists [27].

### 3.6 Completeness of Radiology Reports

Completeness of cross‐sectional imaging reports was assessed in three of the six included studies [23, 24, 27].

Wildman‐Tobriner et al. evaluated 75 SR and 75 NSR for 25 MREs, finding that SR more consistently documented 15 predefined IBD‐related features (mean: 14.0/15 vs. 7.7/15, *p* < 0.05) [24]. In a follow‐up study involving 270 SR and 270 NSR for 30 CTEs, SR again demonstrated higher inclusion of key features (mean: 14.6/15 vs. 8.2/15, *p* < 0.05) [23]. Bailey et al. compared 80 SR and 80 NSR for 30 MRE and 50 CTE and found that 77 out of 80 SR included all 11 essential radiological features, compared to just 40 out of 80 NSR [27].

## 4 Discussion

This systematic review is the first to evaluate the radiological reporting preferences of IBD clinicians in the context of IBD‐related imaging. Across six observational studies, SR was consistently preferred over NSR in key domains, including clarity, clinical utility, and report completeness (Table 3). The findings across these studies demonstrated a broadly consistent pattern, reinforcing perceived advantages of SR in facilitating clearer and more actionable reporting for clinical decision‐making.

**TABLE 3:** Comparison of structured and non‐structured radiology reporting in inflammatory bowel disease.

Outcome	SR versus NSR
*Primary outcome: Referring clinician perception*	
Clarity	Improved by SR in most studies [23, 26, 27, 28]; neutral in one study [24]
Ease of information extraction	Improved by SR in all studies [23, 24, 27]
Ability to identify disease activity	Improved by SR in all studies [23, 24, 25, 26]
Impact on clinical decision‐making	SR led to more management changes [25, 27]
Secondary outcome	
Report completeness	Improved by SR in all studies [23, 24, 27]

Abbreviations: NSR, non‐structured reporting; SR, structured reporting.

Perceived clarity, evaluated in five of the six studies, was the most frequently assessed domain [23, 24, 26, 27, 28]. Four studies reported SR to be significantly clearer than NSR in conveying key radiological findings [24, 26, 27, 28]. These findings suggest that SR may enhance the readability and interpretability of radiology reports. However, the absence of standardized definitions for “clarity” across studies limits direct comparisons. Similarly, all three studies that evaluated ease of information extraction reported that referring clinicians preferred SR over NSR formats, indicating that structured formats may facilitate more effective communication, potentially reducing the time and effort required by IBD clinicians to assimilate key radiological findings [23, 24, 27]. Clinicians also frequently favored SR templates in terms of clinical utility, reflected by clinicians indicating that SR improved their confidence in the detection of active, stricturing, and penetrating Crohn's disease—findings directly relevant to clinical decision‐making. Furthermore, two studies assessed the impact of SR on clinical decision‐making, both finding that SR led to more frequent changes to clinical management plans than NSR [25, 27]. However, these studies were of moderate quality, involved only five clinicians across two institutions, and did not assess whether these changes improved patient outcomes, limiting generalizability and illuminating the need for more robust research to determine whether SR templates provide objective clinical benefits. Reassuringly, the longitudinal study conducted by Zhu et al. showed that 46 Crohn's disease patients treated under a tailored regime using information from SR had improved patient‐reported outcomes with higher quality of life, compared to 46 Crohn's disease patients treated with standard care (inflammatory bowel disease questionnaire total score median 178.07 vs. 162.80, *p* < 0.05) [28]. Collectively, these results indicate that referring IBD clinicians prefer SR over NSR radiological reporting formats; however, these findings are based on small and underpowered sample sizes, limiting their generalizability.

A key finding of this systematic review is the potential role of SR in optimizing clinical management of IBD. Two studies assessed this outcome and found that referring clinicians were more likely to initiate or adjust treatments, such as corticosteroids and biologics, when using SR rather than NSR [25, 27]. The reasons for this are likely multifactorial. SR may have conveyed clinically relevant information more clearly and comprehensively, thereby facilitating a better understanding of disease activity, which may have enhanced confidence in clinical decision‐making. Notably, Zhu et al. also reported improved patient‐reported quality of life with SR in a longitudinal study. These findings suggest that SR not only aligns with clinician preferences but may also support more effective patient management and potentially improve outcomes. However, the generalizability of these findings is limited by the small number of studies and lack of real‐world evaluations directly comparing the clinical impact of SR versus NSR in routine IBD care.

Despite support from consensus guidelines such as SAR CDFP‐SPR‐AGA and ECCO‐ESGAR, the adoption of SR in IBD imaging remains inconsistent [16, 17, 18, 19]. Of the six studies included, only four referenced consensus guidelines in their reporting templates, and none adhered fully to the itemized reporting recommendations [23, 24, 25, 28]. Three of the included studies objectively evaluated report completeness, demonstrating that SR templates more consistently included key IBD‐related features than NSR, suggesting that SR may minimize the omission of important radiological findings [23, 24, 27]. This aligns with the documented benefits of SR templates in other conditions, including pelvic endometriosis, intracranial tumors, rectal and pancreatic cancers, and multiple sclerosis [30, 31, 32, 33, 34, 35]. Most SR templates, designed for MRE and CTE, were adapted from the SAR CDFP‐SPR‐AGA consensus guidelines and included key mural and extramural features such as bowel wall thickening, mucosal enhancement, the comb sign, fibrofatty proliferation, and complications like strictures and fistulas (Table 4) [23, 24, 25, 28]. Radiological assessments such as disease location, disease length, and presence of perianal disease were also frequently reported; however, other radiological features, including perforation, bowel motility, diffusion restriction, and assessments of therapeutic response, were infrequently incorporated despite guideline recommendations. This highlights the need to align SR templates with society‐endorsed guidelines, clearly distinguishing essential from optional reporting parameters.

**TABLE 4:** Radiological parameters included in structured reporting templates in inflammatory bowel disease.

	Wildman‐Tobriner et al. (2017) [24]	Wildman‐Tobriner et al. (2017) [23]	Chiplunker et al. (2018) [25]	Gomez et al. (2018) [26]	Bailey et al. (2021) [27]	Zhu et al. (2024) [28]
Indication	Pediatric Crohn's disease	IBD	Crohn's disease	IBD	IBD	Crohn's disease
Imaging modality	MRE	CTE	MRE	CTE	MRE, CTE	CTE
*Mural*						
Thickening	✓	✓	✓	✓	✓	✓
Enhancement pattern	✓	✓	✓			✓
Ulceration			✓		✓	
Oedema			✓			
Stratification				✓		
Permanent structural changes				✓		
Other findings						✓
*Extra‐mural*						
Comb sign	✓	✓	✓	✓	✓	
Fibrofatty proliferation	✓	✓		✓	✓	
Mesenteric lymphadenopathy	✓	✓	✓	✓		
Fluid collection	✓	✓				
Mesenteric oedema			✓		✓	
Mesenteric related findings						✓
*Extra‐intestinal*						
Sacroiliitis	✓	✓				
Extra‐intestinal findings					✓	✓
*Disease complications*						
Stricture	✓	✓	✓	✓	✓	
Fistula	✓	✓		✓	✓	
Abscess			✓	✓	✓	
Stricture upstream dilatation			✓	✓	✓	
Perforation				✓		
Toxic megacolon				✓		
Inflammatory mass					✓	
Sinus tract					✓	
Penetrating lesions						✓
Complications						✓
Other						
Disease location	✓	✓	✓		✓	✓
Disease length	✓	✓	✓		✓	
Perianal disease	✓	✓			✓	✓
Prior surgery	✓	✓				
Low density fecal material	✓	✓				
Avascular necrosis	✓	✓				
Diffusion restriction			✓			
MEGS			✓			
Colorectal cancer				✓		
Number of disease segments					✓	
Inflammatory changes					✓	
Prior imaging comparison						✓

Abbreviations: CTE, computed tomography enterography; ✓ radiological parameters included in the final scoring formula; MEGS, magnetic resonance imaging enterography global score; MRE, magnetic resonance enterography.

It is also important to consider radiologist perspectives on SR in the context of IBD. A recent survey of Italian radiologists found that approximately one‐third routinely employed SR for MRE in IBD, recognizing its benefits in enhancing report clarity, completeness, and communication with referring clinicians [36]. However, concerns were raised regarding decreased flexibility and increased time requirements. Adoption was notably higher in university hospitals and high‐volume centers. These findings underscore the need for targeted strategies to overcome barriers and promote broader uptake across diverse clinical settings. In this regard, the integration of large language models (LLMs) and artificial intelligence (AI) into structured reporting may hold significant promise, particularly in improving accuracy, efficiency, and standardization of SR. A recent study demonstrated that LLM‐based extraction of imaging features from radiology reports can effectively automate disease activity scoring in Crohn's disease with high accuracy, reducing manual reporting time and enhancing consistency [37]. Such AI‐driven tools have the potential to address many of the current challenges faced by radiologists and support more objective assessment of disease activity.

Several limitations of this systematic review should be acknowledged. First, only six studies met the inclusion criteria, most of moderate quality, involving a total of 19 referring clinicians from medical and surgical specialties. The small number of eligible studies and the limited sample of clinicians restrict the generalizability of the findings to broader IBD clinician populations and illuminate the potential for publication bias. Moreover, the uneven representation of medical and surgical clinicians limits insights into the role of SR in surgical decision‐making. These factors underscore the need for broader stakeholder engagement and more representative sampling in future research. Second, the composite primary outcomes of perceived clarity and clinical utility were subjective and seldom explicitly defined. Although SR was comparably defined in five of six studies, NSR was only defined in half. Collectively, these inconsistencies highlight the need to standardize outcomes and reporting definitions in future research. Third, none of the included studies evaluated SR in pelvic MRI or IUS, both of which are routinely used in IBD care. IUS is frequently performed by gastroenterologists, who directly report and integrate findings into clinical practice. It would therefore be valuable to explore how reporting practices differ between gastroenterologists and radiologists in IBD. The communication of radiological findings in complex perianal Crohn's disease on pelvic MRI represents another important and clinically relevant area. A study by Tuncyurek et al. exemplified this importance by comparing SR and NSR of pelvic MRI in 105 patients (47 with IBD) and found SR provided greater clarity (mean 7.1–8.3 vs. 5.2–7.6), surgical planning utility (7.1–7.6 vs. 5.8–7.1), and report completeness (81.3%–88.3% vs. 61.2%–72.5%; all *p* ≤ 0.05) [38]. However, it was excluded from this review because outcomes for Crohn's disease could not be distinguished from those of non‐IBD patients. A major methodological limitation across all included studies was the inconsistent definition of key outcomes such as “clarity” and “clinical utility.” Additionally, NSR was poorly defined in several studies, further limiting comparability. Future research should adopt standardized definitions for SR and NSR to enable more consistent and meaningful evaluation. There is also limited evidence comparing the interobserver variability in SR and NSR for IBD, which may serve as an important impetus to review and improve current reporting practices. Finally, the retrospective nature of several of the included studies precludes definitive conclusions about causality between SR and observed outcomes.

The current ECCO‐ESGAR guidelines encourage, but do not mandate, the use of validated radiological disease activity scores and standardized reporting templates in IBD care [2, 16]. This variability in adoption was reflected in the included studies, with only one incorporating a radiological disease activity score into the SR template [25]. Similarly, radiologist perspectives, particularly regarding the impact of SR on workload and reporting time, were underexplored. Only one included study assessed radiologist opinions, reporting mostly positive (44%) or neutral (44%) views towards SR in IBD reporting [23]. In addition, a separate study found that radiologists preferred SR over NSR for small bowel imaging using CT, as demonstrated by significantly higher satisfaction scores (4.0–4.1 vs. 2.6–3.2; *p* < 0.05) [39]. These findings suggest a potential preference among radiologists for SR; however, small sample sizes and limited representation restrict generalizability and highlight the need for larger, targeted surveys of radiologists involved in IBD care.

## 5 Conclusion and Future Directions

Effective communication between radiologists and IBD clinicians is essential to support optimal clinical decision‐making. This systematic review highlights the potential for SR to enhance interdisciplinary communication and standardize the presentation of key radiological findings in IBD. This is particularly relevant in Crohn's disease, where MRI plays a central role in evaluating complex disease, monitoring interval changes, and assessing treatment response. As radiological treatment targets for both luminal and perianal Crohn's disease become more clearly defined, the need for clear, consistent, and actionable imaging reports is likely to increase. Fostering a productive and collaborative relationship between radiologists and IBD clinicians remains critical to ensuring that reports are not only comprehensive and standardized but also nuanced and clinically relevant. However, current evidence comparing SR with NSR is limited by methodological heterogeneity, variation in reporting templates, and a lack of high‐quality prospective studies. Notably, none of the included studies evaluated SR in the context of pelvic MRI or IUS, both of which are important and frequently used investigations for the assessment of Crohn's disease. Future research should prioritize prospective, real‐world evaluations of SR in IBD, with particular attention to clinical impact, reporting efficiency, interobserver variability, and the integration of AI. Efforts to align reporting templates with expert consensus guidelines and to integrate radiologist perspectives will be essential for supporting wider implementation and sustained clinical utility.

## Conflicts of Interest

A.V. has served on the advisory boards of Abbvie, Pfizer, and Ferring, and has served as a speaker for Abbvie and Pfizer. A.S. has served as a speaker for Arrotex Pharmaceuticals, received educational support from Dr. Falk Pharma, and received advisory fees from AstraZeneca, AbbVie, and Takeda Pharmaceuticals. The other authors declare no conflicts of interest.

## Supporting information


**Table S1:** Database search strategy.
**Table S2:** Risk of bias assessment of each study using the Joanna Briggs Institute critical appraisal tool.

## Data Availability

The data that support the findings of this study are available from the corresponding author upon reasonable request.

## References

[1] C. A. Puylaert , J. A. Tielbeek , S. Bipat , and J. Stoker , “Grading of Crohn's Disease Activity Using CT, MRI, US and Scintigraphy: A Meta‐Analysis,” European Radiology 25, no. 11 (2015): 3295–3313, 10.1007/s00330-015-3737-9. 26080794 PMC4595539

[2] R. W. Lo , G. Bhatnagar , N. Kutaiba , and A. R. Srinivasan , “Evaluating Luminal and Post‐Operative Crohn's Disease Activity on Magnetic Resonance Enterography: A Review of Radiological Disease Activity Scores,” World Journal of Gastroenterology 31, no. 26 (2025): 107419. 40678704 10.3748/wjg.v31.i26.107419 PMC12264814

[3] H. Habeeb , L. Chen , I. De Kock , et al., “Imaging in Perianal Fistulising Crohn's Disease: A Practical Guide for the Gastroenterologist,” World Journal of Gastroenterology 31, no. 34 (2025): 110611. 40937454 10.3748/wjg.v31.i34.110611 PMC12421400

[4] S. S. Naik , A. Hanbidge , and S. R. Wilson , “Radiology Reports: Examining Radiologist and Clinician Preferences Regarding Style and Content,” American Journal of Roentgenology 176, no. 3 (2001): 591–598, 10.2214/ajr.176.3.1760591. 11222186

[5] B. Kowalczyk , P. Ramis , A. Hillman , et al., “Radiology Reporting Preferences: What Do Referring Clinicians Want?,” Academic Radiology 32, no. 1 (2025): 439–449, 10.1016/j.acra.2024.09.006. 39299861

[6] P. A. Marcovici and G. A. Taylor , “Journal Club: Structured Radiology Reports Are More Complete and More Effective Than Unstructured Reports,” AJR. American Journal of Roentgenology 203, no. 6 (2014): 1265–1271, 10.2214/AJR.14.12636. 25415704

[7] B. D. Jackson , D. Con , A. Gorelik , D. Liew , S. Knowles , and P. De Cruz , “Examination of the Relationship Between Disease Activity and Patient‐Reported Outcome Measures in an Inflammatory Bowel Disease Cohort,” Internal Medicine Journal 48, no. 10 (2018): 1234–1241, 10.1111/imj.13937. 29663629

[8] D. B. Larson , A. J. Towbin , R. M. Pryor , and L. F. Donnelly , “Improving Consistency in Radiology Reporting Through the Use of Department‐Wide Standardized Structured Reporting,” Radiology 267, no. 1 (2013): 240–250, 10.1148/radiol.12121502. 23329657

[9] L. H. Schwartz , D. M. Panicek , A. R. Berk , Y. Li , and H. Hricak , “Improving Communication of Diagnostic Radiology Findings Through Structured Reporting,” Radiology 260, no. 1 (2011): 174–181, 10.1148/radiol.11101913. 21518775 PMC3121011

[10] V. Granata , R. Grassi , V. Miele , et al., “Structured Reporting of Lung Cancer Staging: A Consensus Proposal,” Diagnostics (Basel, Switzerland) 11, no. 9 (2021): 1569, 10.3390/diagnostics11091569. 34573911 PMC8465460

[11] V. Granata , D. Caruso , R. Grassi , et al., “Structured Reporting of Rectal Cancer Staging and Restaging: A Consensus Proposal,” Cancers (Basel) 13, no. 9 (2021): 1–33, 10.3390/cancers13092135. PMC8125446 33925250

[12] D. L. Weiss and C. P. Langlotz , “Structured Reporting: Patient Care Enhancement or Productivity Nightmare?,” Radiology 249, no. 3 (2008): 739–747, 10.1148/radiol.2493080988. 19011178

[13] J. M. Nobel , K. van Geel , and S. G. F. Robben , “Structured Reporting in Radiology: A Systematic Review to Explore Its Potential,” European Radiology 32, no. 4 (2022): 2837–2854, 10.1007/s00330-021-08327-5. 34652520 PMC8921035

[14] A. J. Johnson , M. Y. Chen , J. S. Swan , K. E. Applegate , and B. Littenberg , “Cohort Study of Structured Reporting Compared With Conventional Dictation,” Radiology 253, no. 1 (2009): 74–80, 10.1148/radiol.2531090138. 19709993

[15] A. J. Johnson , M. Y. Chen , M. E. Zapadka , E. M. Lyders , and B. Littenberg , “Radiology Report Clarity: A Cohort Study of Structured Reporting Compared With Conventional Dictation,” Journal of the American College of Radiology 7, no. 7 (2010): 501–506, 10.1016/j.jacr.2010.02.008. 20630384

[16] T. Kucharzik , J. Tielbeek , D. Carter , et al., “ECCO‐ESGAR Topical Review on Optimizing Reporting for Cross‐Sectional Imaging in Inflammatory Bowel Disease,” Journal of Crohn's and Colitis 16, no. 4 (2022): 523–543, 10.1093/ecco-jcc/jjab180. 34628504

[17] F. F. Guglielmo , S. A. Anupindi , J. G. Fletcher , et al., “Small Bowel Crohn Disease at CT and MR Enterography: Imaging Atlas and Glossary of Terms,” Radiographics 40, no. 2 (2020): 354–375, 10.1148/rg.2020190091. 31951512

[18] M. E. Baker , A. K. Hara , J. F. Platt , D. D. Maglinte , and J. G. Fletcher , “CT Enterography for Crohn's Disease: Optimal Technique and Imaging Issues,” Abdominal Imaging 40, no. 5 (2015): 938–952, 10.1007/s00261-015-0357-4. 25637126

[19] D. H. Bruining , E. M. Zimmermann , E. V. Loftus, Jr. , et al., “Consensus Recommendations for Evaluation, Interpretation, and Utilization of Computed Tomography and Magnetic Resonance Enterography in Patients With Small Bowel Crohn's Disease,” Radiology 286, no. 3 (2018): 776–799, 10.1148/radiol.2018171737. 29319414

[20] M. J. Page , J. E. McKenzie , P. M. Bossuyt , et al., “The PRISMA 2020 Statement: An Updated Guideline for Reporting Systematic Reviews,” BMJ (Clinical Research Edition) 372 (2021): n71, 10.1136/bmj.n71. PMC8005924 33782057

[21] Veritas Health Innovation M, Australia , “Covidence Systematic Review Software,” 2025.

[22] S. M. Z. Moola , C. Tufanaru , E. Aromataris , et al., “Chapter 7: Systematic Reviews of Etiology and Risk in,” in JBI Manual for Evidence Synthesis, ed. E. M. Z. Aromataris (JBI, 2020).

[23] B. Wildman‐Tobriner , B. C. Allen , M. R. Bashir , et al., “Structured Reporting of CT Enterography for Inflammatory Bowel Disease: Effect on Key Feature Reporting, Accuracy Across Training Levels, and Subjective Assessment of Disease by Referring Physicians,” Abdominal Radiology 42, no. 9 (2017): 2243–2250, 10.1007/s00261-017-1136-1. 28393301

[24] B. Wildman‐Tobriner , B. C. Allen , J. T. Davis , et al., “Structured Reporting of Magnetic Resonance Enterography for Pediatric Crohn's Disease: Effect on Key Feature Reporting and Subjective Assessment of Disease by Referring Physicians,” Current Problems in Diagnostic Radiology 46, no. 2 (2017): 110–114, 10.1067/j.cpradiol.2016.12.001. 28062089

[25] A. Chiplunker , R. Tsai , G. Christophi , et al., “Impact of a Standardized Reporting System for Magnetic Resonance Enterography on Small Bowel Crohn's Disease Management 575,” American Journal of Gastroenterology 113 (2018): S331, 10.14309/00000434-201810001-00575.

[26] A. A. Gomez , T. F. Nunes , C. H. M. Santos , et al., “Comparison of Conventional and Structured Report in the Evaluation of Crohn's Disease Through Enterography,” Journal of Coloproctology 38, no. 4 (2018): 290–294, 10.1016/j.jcol.2018.05.011.

[27] R. Bailey , F. A. Farraye , M. Picco , J. Cernigliaro , and C. Bolan , “A Quality Improvement Program to Standardize Enterography Reports for Patients With Inflammatory Bowel Disease S925,” American Journal of Gastroenterology 116 (2021): S437–S438, 10.14309/01.ajg.0000777232.08902.18.

[28] H. Zhu , S. Chen , J. Chen , et al., “Structured Reporting of Computed Tomography Enterography in Crohn's Disease,” Current Medical Imaging 20 (2024): e15734056258848, 10.2174/0115734056258848240101055747. 38310552

[29] P. S. Panizza , P. C. Viana , N. Horvat , et al., “Inflammatory Bowel Disease: Current Role of Imaging in Diagnosis and Detection of Complications: Gastrointestinal Imaging,” Radiographics 37, no. 2 (2017): 701–702, 10.1148/rg.2017160050. 28287943

[30] C. C. Barbisan , M. P. Andres , L. R. Torres , et al., “Structured MRI Reporting Increases Completeness of Radiological Reports and Requesting Physicians' Satisfaction in the Diagnostic Workup for Pelvic Endometriosis,” Abdominal Radiology 46, no. 7 (2021): 3342–3353, 10.1007/s00261-021-02966-4. 33625575

[31] A. Bink , J. Benner , J. Reinhardt , et al., “Structured Reporting in Neuroradiology: Intracranial Tumors,” Frontiers in Neurology 9 (2018): 32, 10.3389/fneur.2018.00032. 29467712 PMC5808104

[32] J. J. C. Tersteeg , P. D. Gobardhan , R. Crolla , et al., “Improving the Quality of MRI Reports of Preoperative Patients With Rectal Cancer: Effect of National Guidelines and Structured Reporting,” AJR. American Journal of Roentgenology 210, no. 6 (2018): 1240–1244, 10.2214/AJR.17.19054. 29570375

[33] A. Franconeri , J. Fang , B. Carney , et al., “Structured vs Narrative Reporting of Pelvic MRI for Fibroids: Clarity and Impact on Treatment Planning,” European Radiology 28, no. 7 (2018): 3009–3017, 10.1007/s00330-017-5161-9. 29247353

[34] E. Dickerson , M. S. Davenport , F. Syed , et al., “Effect of Template Reporting of Brain MRIs for Multiple Sclerosis on Report Thoroughness and Neurologist‐Rated Quality: Results of a Prospective Quality Improvement Project,” Journal of the American College of Radiology 14, no. 3 (2017): 371, e371–379, 10.1016/j.jacr.2016.09.037. 27932248

[35] O. R. Brook , A. Brook , C. M. Vollmer , T. S. Kent , N. Sanchez , and I. Pedrosa , “Structured Reporting of Multiphasic CT for Pancreatic Cancer: Potential Effect on Staging and Surgical Planning,” Radiology 274, no. 2 (2015): 464–472, 10.1148/radiol.14140206. 25286323

[36] C. Bonifacio , A. Dal Buono , R. Levi , et al., “Reporting of Magnetic Resonance Enterography in Inflammatory Bowel Disease: Results of an Italian Survey,” Journal of Clinical Medicine 13, no. 13 (2024): 3953. 38999518 10.3390/jcm13133953 PMC11242042

[37] R. Dehdab , F. Mankertz , J. M. Brendel , et al., “LLM‐Based Extraction of Imaging Features From Radiology Reports: Automating Disease Activity Scoring in Crohn's Disease,” Academic Radiology (forthcoming). 10.1016/j.acra.2025.07.041 40783343

[38] O. Tuncyurek , A. Garces‐Descovich , A. Jaramillo‐Cardoso , et al., “Structured Versus Narrative Reporting of Pelvic MRI in Perianal Fistulizing Disease: Impact on Clarity, Completeness, and Surgical Planning,” Abdominal Radiology 44, no. 3 (2019): 811–820, 10.1007/s00261-018-1858-8. 30519819

[39] J. Chen , J. Zhou , S. Gong , et al., “Impact of a Structured Report Template on the Quality of Multislice Spiral Computed Tomography Scan Reports for Small Bowel Diseases: A Preliminary Study,” Iranian Journal of Radiology 19, no. 3 (2022): 1–10.

