# Supplementary material for: Structured Versus Non‐Structured Reporting of Inflammatory Bowel Disease Imaging: A Systematic Review
Source: JGH Open. 2025 Sep 28;9(10):e70288. doi: 10.1002/jgh3.70288 (PMC12477271; doi:10.1002/jgh3.70288)
Supplement: Supplementary file 1 — Table S1: Database search strategy. Table S2: Risk of bias assessment of each study using the Joanna Briggs Institute critical appraisal tool. [file JGH3-9-e70288-s001.docx]

**Supplementary Material**

**Supplementary Table 1.** **Database search strategy.**

| **EMBASE, MEDLINE, CENTRAL (OVID)** | |
| --- | --- |
| **#** | **Search terms** |
| 1 | exp Inflammatory Bowel Diseases/ |
| 2 | crohn* or ulcerative colitis or perianal fistul* or inflammatory bowel or IBD |
| 3 | 1 or 2 |
| 4 | MRI or magnetic resonance imaging or MRE or enterograph* or IUS or ultrasound or CT or CTE or tomograph* |
| 5 | structure* or standardi* or template* or convention* or narrati* or context* or free text* |
| 6 | report* |
| 7 | 3 and 4 and 5 and 6 |

**Supplementary Table 2.** **Risk of bias assessment of each study using the Joanna Briggs Institute critical appraisal tool.**

|  | **Wildman-Tobriner *et al.* (2017) ^[22]^** | **Wildman-Tobriner *et al.* (2017) ^[21]^** | **Chiplunker *et al.***  **(2018) ^[24]^** | **Gomez *et al.***  **(2018) ^[26]^** | **Bailey *et al.***  **(2021) ^[23]^** | **Zhu *et al.***  **(2024) ^[25]^** |
| --- | --- | --- | --- | --- | --- | --- |
| **1. Is it clear in the study what is the “cause” and what is the “effect”?** | **✓** | **✓** | **✓** | **✓** | **✓** | **✓** |
| **2. Was there a control group?** | **✓** | **✓** | **✓** | **✓** | **✓** | **✓** |
| **3. Were participants included in any comparisons similar?** | **✓** | **✓** | X | X | X | X |
| **4. Were the participants included in any comparisons receiving similar treatment/care, other than the exposure or intervention of interest?** | **✓** | **✓** | X | X | X | X |
| **5. Were there multiple measurements of the outcome, both pre and post the intervention/exposure?** | **✓** | **✓** | **✓** | **✓** | **✓** | **✓** |
| **6. Were the outcomes of participants included in any comparisons measured in the same way?** | **✓** | **✓** | **✓** | **✓** | **✓** | **✓** |
| **7. Were outcomes measured in a reliable way?** | X | X | X | X | X | X |
| **8. Was follow-up complete and if not, were differences between groups in terms of their follow-up adequately described and analysed?** | N/A* | N/A* | N/A* | N/A* | N/A* | X |
| **9. Was appropriate statistical analysis used?** | **✓** | **✓** | **✓** | **✓** | ? | **✓** |
| **Overall score** | 7/8 | 7/8 | 5/8 | 5/8 | 4/8 | 5/9 |
| **Quality appraisal** | High | High | Moderate | Moderate | Moderate | Moderate |

*N/A: not applicable due to not being a longitudinal study
